# Minority Stress, Caregiving Experiences, and Health of Bi+ Informal Caregivers of People with Alzheimer’s Disease and Related Dementias

**DOI:** 10.1002/alz.091581

**Published:** 2025-01-09

**Authors:** Krystal R. Kittle, Ethan C. Cicero, Jordan Pelkmans, Jason D. Flatt, Joel G Anderson

**Affiliations:** ^1^ University of Massachusetts‐Amherst, Amherst, MA USA; ^2^ Emory University, Atlanta, GA USA; ^3^ University of Nevada Las Vegas School of Public Health, Las Vegas, NV USA; ^4^ College of Nursing, University of Tennessee, Knoxville, TN USA

## Abstract

**Background:**

Bi+ people, those with non‐monosexual identities (e.g., bisexual, pansexual, sexually fluid) are more likely to experience poor physical and mental health compared with monosexual minority (i.e., gay[G], lesbian[L]) people. These poor health outcomes stem from minority stress related to their sexual minority status (e.g., discrimination, victimization). Due to minority and caregiver stress, LGB+ people providing care to someone with ADRD are more likely to have worse health than their straight counterparts. Little is known about how minority and caregiver stress affect the health of bi+ ADRD caregivers versus monosexual minority ADRD caregivers.

**Method:**

Cross‐sectional survey data from a non‐probabilistic sample of sexual minority ADRD caregivers (bi+, *n* = 125; gay/lesbian, *n* = 161) were used to examine associations between minority stress (discrimination, victimization, microaggressions, perceived stress), caregiver stress (caring for spouse/partner, respite use, distress from care recipient’s neuropsychiatric symptoms, care recipient’s level of independence) on overall self‐rated health and if these associations differed among bi+ and monosexual minority ADRD caregivers. Multivariable regression, adjusting for demographic characteristics, was used to investigate if sexual orientation (bi+ vs. gay/lesbian) moderated the association between minority/caregiver stress variables and health.

**Result:**

All minority stressors and two caregiving stressors (caring for spouse, care recipient’s level of independence) were associated with overall health. In our adjusted model, three variables (lifetime victimization, day‐to‐day discrimination, caring for spouse/partner) moderated the association between sexual orientation and overall health. As lifetime victimization scores increased, bi+ overall health decreased while monosexual minority overall health increased (*B* = ‐0.56; 95% CI = 1.06,0.05; *p* = 0.03). Caring for a spouse/partner, protective for both groups (*B* = 5.11; 95%CI = 3.04,7.17; *p*<0.001), had a greater effect on health for monosexual minorities (*B* = ‐4.26; 95% CI = ‐7.49,‐1.03; *p*<0.001). Higher day‐to‐day discrimination scores predicted worse health for both groups (*B* = ‐0.61; 95% CI = ‐0.91,‐0.32; *p*<0.001), but the magnitude was greater for monosexual minorities (*B* = 0.41; 95% CI = 0.01,0.80; *p* = 0.044).

**Conclusion:**

The effects of lifetime victimization, day‐to‐day discrimination, and caring for one’s spouse/partner on health differ significantly dependent on being bi+ or gay/lesbian. Qualitative inquiries may help illuminate the underlying mechanisms for this phenomenon, informing interventions and policy development aimed at supporting the health and well‐being of bi+ ADRD caregivers.

